# ﻿Reconnecting research and natural history museums in Italy and the need of a national collection biorepository

**DOI:** 10.3897/zookeys.1104.79823

**Published:** 2022-06-08

**Authors:** Franco Andreone, Ferdinando Boero, Marco A. Bologna, Giuseppe M. Carpaneto, Riccardo Castiglia, Spartaco Gippoliti, Bruno Massa, Alessandro Minelli

**Affiliations:** 1 Museo Regionale di Scienze Naturali, Via G. Giolitti, 36, I-10123 Torino, Italy Museo Regionale di Scienze Naturali Torino Italy; 2 Università di Napoli Federico II, CNR-IAS, Stazione Zoologica Anton Dohrn, Villa Comunale, I-80121 Napoli, Italy Università di Napoli Federico II Napoli Italy; 3 Dipartimento di Scienze, Università Roma Tre, Viale G. Marconi, 446, I-00146 Roma, Italy Università Roma Tre Roma Italy; 4 Dipartimento di Biologia e Biotecnologie “Charles Darwin”, Università “La Sapienza” di Roma, Via A. Borelli, 50, I-00161 Roma, Italy Università “La Sapienza” di Roma Roma Italy; 5 Società Italiana per la Storia della Fauna “Giuseppe Altobello”, Viale Liegi, 48A, I-00198 Roma, Italy Società Italiana per la Storia della Fauna “Giuseppe Altobello” Rome Italy; 6 Dipartimento di Scienze agrarie, alimentari e forestali, Università di Palermo, Viale Scienze, 13, I-90128 Palermo, Italy Università di Palermo Palermo Italy; 7 Dipartimento di Biologia, Università di Padova, Via Ugo Bassi, 58B, I-35131 Padova, Italy Università di Padova Padova Italy

**Keywords:** Biodiversity, biogeography, biorepository, collections, conservation, national natural history museum, taxonomy

## Abstract

In Italy, differently from other countries, a national museum of natural history is not present. This absence is due, among other reasons, to its historical political fragmentation up to 1870, which led to the establishment of medium-sized museums, mostly managed by local administrations or universities. Moreover, a change of paradigm in biological research, at the beginning of the 20^th^ century, contributed to privilege experimental studies in universities and facilitated the dismissal of descriptive and exploratory biology, which formed the basis of the taxonomic research carried out by natural history museums. Consequently, only a few museums have a provision of curatorial staff, space and material resources adequate to maintain their original mission of discovering the natural world, by conducting a regular research activity accompanied by field campaigns. The creation of a national research centre for the study of biodiversity, facilitating interconnections among the existing natural history museums could be a solution and is here supported, together with a centralised biorepository to host collections and vouchers, to the benefit of current and future taxonomic research and environmental conservation. Such an institution should find place and realisation within the recently proposed National Biodiversity Future Center (NBFC) planned within the National Plan of Recovery and Resilience (PNRR). Pending upon the creation of this new national centre, a network among the existing museums should coordinate their activities.

Natural history museums (hereafter “museums”) played and still play a crucial role in the discovery and description of the natural world (Davis 1996; Bakker et al. 2020). Their genesis and development vary largely across countries, mostly depending on a heterogeneous array of historical constraints and geopolitical aspects.

Current museums’ core missions include pivotal activities, among which: (a) gathering, preserving, digitalizing, and implementing scientific collections of natural objects, organisms, and parts of them; (b) exploring and monitoring biodiversity, discovering living beings, describing and interpreting wildlife in taxonomic, biogeographical, and ecological terms; and (c) disseminating scientific knowledge. In particular, museums are prominent hubs of taxonomic studies: with an estimate of eight million eukaryotic species present on Earth—of which barely two million are described today—intensive field surveys and taxonomic assessments are badly needed to fill the gap in the knowledge of our planet’s biodiversity, and, finally, to promote its conservation (Dubois 2003; Boero 2010; Costello et al. 2013). Museums share the primary mission of hosting natural history collections: these often include type series (specimens upon which taxa are described) and voucher series, which are useful to support the knowledge of species, including morphology, distribution, ecology, and evolution.

Many existing scientific collections were assembled by naturalists in the 18^th^ and 19^th^ centuries and, thus, also have a historical importance (Clemann et al. 2014; Camacho et al. 2018). As a general trait, collections form a sound scientific reference for present and future research, including studies to understand and interpret the geographic and genetic variation of the species and populations, as well as the changes in their distribution range. Beside this, museums have the commitment to conserve biodiversity, an objective representing a recent complement to the traditional ones and highlighting how biological conservation can profit from taxonomy, faunal and floral studies, and biogeography (Butler et al. 1998; McCarter et al. 2001; Dubois 2010; Engel et al. 2021). For all these aspects, museums can also be considered true “Alexandrine libraries”, where the knowledge of the natural world is made available to future generations (Funk et al. 2005; Rocha et al. 2014; Salick et al. 2014; Schilthuizen et al. 2015; Dubois 2017; Rohwer et al. 2022).

Sadly, although terms as “biodiversity” and “ecology” are frequently used in daily narrations and political programs, many museums—which should be the places where these ideas and programs are emphasised and made relevant (Massa, 2021)—are currently facing critical difficulties, in particular in assuring a constant census of the planetary diversity and in warranting science dissemination (Alberch et al. 1994). Italy, in particular, is facing serious problems associated with the management of museums, as already highlighted by Andreone et al. (2014).

We believe that the absence of a national institution is particularly critical and associated to the absence of a relevant research activity. We strongly believe that Italy needs a national repository and/or a national centre to assure that taxonomic studies are pursued, together with voucher-collecting activity. In this contribution we provide a historical overview of the Italian situation and make some operative proposals.

## ﻿The birth of Italian natural history museums and the historical background

We here summarise the historical aspects behind the formation of Italian museums, since probably not all readers are aware of the fluid geopolitical subdivision of the Italian territory from the period in which the first natural history collections appeared until Italian unification in 1870.

After the Congress of Vienna (1815), Italy was still divided into several dynastic territories belonging to foreign kingdoms or powerful local houses. Most of northern Italy was split between the Kingdom of Savoy-Sardinia (Piedmont, Liguria, and Sardinia) and Lombardy-Venetia (under the rule of the Austrian Empire), with minor entities such as the Duchy of Parma and Piacenza and the Duchy of Modena and Reggio (both under the rule of the Austrian Empire). Most of central Italy belonged to the State of the Church (from Romagna to Latium) or to the ancient Grand Duchy of Tuscany, while southern Italy was unified under the Kingdom of the Two Sicilies, ruled by the Hispanic branch of the Bourbon dynasty. The official birth of united Italy is dated 1861 but the almost complete union occurred only in 1870 after the Third Independence War and the occupation of Rome and Latium; Rome became the capital in 1871. After the First World War (1918), other north-eastern Italian territories were joined in the Kingdom of Italy.

The long-lasting fragmentation into small states and the lack of a political centralisation until 1870 was accompanied by the birth and affirmation of small to medium-sized natural history museums, together with the evident absence of a large, national museum. Some of the small museums were nevertheless precious for the study of natural diversity at regional level, for example, the natural history museum of Francesco Minà Palumbo (a collection of Sicilian animals, plants, minerals, and fossils he collected and the splendid drawings he painted on the Madonie Mountains from 1837 to 1899), the Royal Mineralogical Museum of Naples (established in 1801), and further collections that gave successively rise to larger natural history museums. Other museums, such as the one in Genoa, or those in Turin and Florence, reached an international importance in the last decades of the 19^th^ century and contributed largely to the advancement of descriptive zoology and botany. In any case, the lack of a unified and independent nation hindered the appearance of a centralised institution for the high-level study of geo- and biodiversity and hampered the development of a nation-wide natural history culture, rarely considered at the same level of humanities, likely also due to the scholastic “Croce-Gentile” reform of around a century ago (Tognon 2016).

Despite this structural deficiency, Italy was the first European country to complete a checklist of its fauna in 1995 (more than 55,000 species listed excluding protozoa; Minelli et al. 1993–1995), with numerous amendments and additions since then (Stoch et al. 2004) showing how provisional our knowledge of biodiversity is. A new online “Checklist” of Metazoa, including some 60,000 species, is now under construction (Bologna et al. 2022).

In other European countries (e.g., Austria, Belgium, Denmark, France, Spain, Sweden, and the United Kingdom), the existing national natural history museums always maintained relevant scientific activities. Other institutions, like the Naturalis Biodiversity Center in Leiden and the Museum für Naturkunde in Berlin, upgraded to new-concept popularization and biodiversity research hubs. In the New World too, the task of making collections and having care of them remained crucial for scientific research in museums. For instance, in the USA (and the whole Western Hemisphere) the American Mammalogical Society, through a Systematic Collections Committee, maintains a census of mammal specimens in qualified institutions to keep high standards of curation (Phillips et al. 2019), standards that are very far from the present Italian situation (Gippoliti et al. 2014). A similar situation is also present in Brazil, where the natural history collections are concentrated in three main national repositories (Museu Nacional - Universidade Federal do Rio de Janeiro, Museu de Zoologia da Universidade de São Paulo, and Museu Paraense Emilio Goeldi), but there are dozens of other natural history collections, mainly associated to universities (Bezerra 2012).

Whereas in the 19^th^ century many Italian natural history museums propelled activities worldwide in the wake of positivism, the inspiration for taxonomic and exploratory studies suddenly faded at the beginning of the new century, despite the colonial research undertaken especially by museums in Genoa, Florence, and Milan (Gippoliti 2005; Chiozzi 2013; Poggi 2017). Thus, museums were increasingly seen as places for science education and outreach, but less as independent research centres. Furthermore, being mostly managed by local administrations (municipalities, regions) or universities, they did not benefit from autonomy and had difficulties in accessing national or international funds. University institutes too often forgot their connections with natural history museums and collections, and/or considered them as useless for modern experimental research. Due to this lack of interest, many universities also failed to care for the maintenance of systematic collections, often seen as obsolete forms of science.

Within this scenario we may already spot some of the difficulties experienced by Italian museums and herbaria in the new millennium: unable to coalesce into larger institutional networks, they were increasingly constrained by limited budgets, lack of space, and reduced personnel. For these reasons, biological surveys, voucher collecting, and collection purchase (which were instead priorities in museums of the 19^th^ century) became rarer. At best museums survived as places where to preserve historical collections; when space was available, a few private collections were also accepted after their owners’ death. In some cases, the historical scientific collections were neglected or abandoned, a circumstance sadly shared by other European countries too (Krištufek et al. 2015; Ceríaco et al. 2021).

## ﻿Research is a major engine for natural history museums

Due to the above-mentioned causes many Italian museums have difficulties playing the role of scientific institutions. In this aspect they differ from larger European museums, which are dedicated biodiversity centres and actively support research in their country or in biodiversity hotspots. Indeed, collection-based research is crucial to perform taxonomic studies, to confirm the presence of a species at well-defined sites, and to assess conservation status or the dynamics of a species’ range, as well as to provide material for the study of some biological traits, such as fecundity and longevity (Tessa et al. 2009). The study of collections also serves to document and unveil life-history parameters and temporal patterns in evolutionary and ecological studies (Wandeler et al. 2007; English et al. 2018). This is, for example, evident when DNA analysis or X-ray CT scans are used to unveil taxonomic and functional aspects (Betz et al. 2007; Broeckhoven and du Plessis 2018). Unfortunately, in Italy almost none of the existing museums have resources to conduct such activities independently and, thus, usually rely on collaborations with other institutions.

The fact that many Italian museums do not have research as a topic mission and do not have a national breadth is also one of the causes for their absence from the European Commission-funded SYNTHESYS+ project, a program creating an integrated European infrastructure for natural history collections (Bartolozzi 2013). In recent times, collections were also used as useful tools to investigate pollution and epidemiology: frozen tissues housed in museums allowed for the discovery of many new hantaviruses in rodents worldwide (Yanagihara et al. 2014; Dunnum et al. 2017) and were used to elucidate the taxonomy of species involved in zoonoses, including the recently widespread SARS-Cov-2 (Colella et al. 2021).

## ﻿Italy’s quest for a national museum

The historical reasons for the lack of a national museum in Italy have already been addressed by Ruffo (2006) and, more recently, by Canadelli (2015), among others, showing that these are mostly associated with Italy’s past political fragmentation. As we saw, only a few pre-unitarian states were able to build sufficiently large museums carrying out regular research activity (Bologna 2015). In Germany, a country with a similar history of political fragmentation, museums generally followed another path. From the beginning, they were classified as research centres and usually collaborated with universities acting as scientific centres or featured a good level of autonomy. Differently from what happened in the Netherlands with the Naturalis Biodiversity Center, which centralised all the country natural history collections, several German museums became associated within the Senckenberg Gesellschaft für Naturforschung and operate as a geographically distributed system of collections.

Sadly, while Italian museums in the 19^th^ century were also highly productive in the context of their educational and/or scientific aims, and often produced prestigious taxonomic and biogeographical schools, they were not able to upgrade later on. Moreover, a strong connection with their hosting town often resulted in a general difficulty and/or unwillingness to give birth to a large institution with national and international scope.

Despite these difficulties, the project of a national museum and/or the implementation of national collections was never forgotten. Many naturalists of the past considered this an important step towards a satisfactory level of natural history studies. In many cases there were concurrent attempts to develop national repositories for natural history collections. As an example, the botanist Filippo Parlatore sent a letter from London on the occasion of the “Terza Riunione degli Scienziati italiani” (Third Meeting of the Italian Scientists, Florence, 15–29 September 1841), suggesting the creation of a “General Herbarium for the Italian Peninsula”, as done by other European countries, proposing Florence as the seat of the “Erbario centrale Italiano” (Capanna 2011). In 1861, just after the unification of Italy, the zoologist Enrico Hyllier Giglioli did his best to put together the first cell of a national museum by establishing in 1877 (again in Florence) the “Collezione Nazionale dei Vertebrati” (National Vertebrate Collection). In 1913, a royal decree established the “Collezione elmintologica centrale italiana e Laboratorio di Elmintologia” (Central Italian helminthological collection and helminthology laboratory) at the Zoological Museum of Naples “Federico II” University. The helminthological collection was set up together with the material assembled by Corrado Parona, Michele Stossich, and Francesco Saverio Monticelli (at that time director, as university professor, of the Zoological Museum), and specifically received financial support from the government to support a curator job position. As a last project of “nationalization” of biological collections, we mention the “Istituto Nazionale di Entomologia” (National Entomological Institute), founded in 1940 by Federico Hartig (1900–1980) and established in Rome, destined to become in 1977 an important part of a new museum of zoology at “La Sapienza” University (Vigna Taglianti and Zilli 2008).

## ﻿A proposal for a centralised biorepository

It is, therefore, remarkable that in the last 80 years, while biodiversity has increasingly become a global scientific issue, no real attempt has yet been made to foster collection-based biodiversity research, except for a failed attempt in the 1980s to organize a national museum in Florence, pursued by the Accademia Nazionale dei Lincei (Canadelli 2015).

The position of the Italian Peninsula—at the centre of the Mediterranean region, acting as a geographic bridge between Europe and Africa, and having complex palaeogeographic history—and its importance as repeated glacial refuges make research on Italian marine and land ecosystems of international importance (Giaccone et al. 2008), given also that Italy has the richest biodiversity in the European Union (Bologna et al. 2022). In addition, Italian museums host important collections from all over the world, a precious heritage of travellers and scientists who had a global impact on biodiversity research (Pichi-Sermolli 1988; Gippoliti 2005). Aside from those collected in former Italian colonies in Africa, we also mention the rich zoological collections from Borneo (Peters 1872) and New Guinea (Peters and Doria 1878), both housed in the Genoa Museum; the collections from Latin America collected in the 19^th^ century by Alfredo Borelli and Enrico Festa, and in the 20^th^ century by Josè M. Cei in the Turin Museum (Lavilla et al. 2008). Yet, the present fragmentation of museums and associated collections does not allow for an effective participation of Italy to global models of aggregated natural history databases, such as the VertNet for vertebrates (http://www.vertnet.org), the Integrated Digitized Biocollections (iDigBio, https://www.idigbio.org/), and the Global Biodiversity Information Facility (GBIF, https://www.gbif.org/), toward which museums are moving worldwide (Minelli 2015).

In the light of the difficulties experienced in the past to build a national museum in Italy, we wonder whether there is still room for such a proposal, or, instead, we need to develop a new concept of centralisation and connection. The “Centro Nazionale per la Biodiversità” (NBFC: National Biodiversity Future Centre) planned within the initiatives of the Italian “Piano Nazionale di Ripresa e Resilienza” (PNRR: National Plan of Recovery and Resilience) has the great potential to offer a unique occasion to boost the establishment of a national institution dedicated to biodiversity, taxonomy, and conservation (Ferrari 2022).

Given the crucial role played nowadays by museums for the study, popularisation, and conservation of biodiversity, the realization of a centralised repository is an urgent logical step, especially in the light of a novel awareness of the importance of both biodiversity and ecosystems, as recognized by the European Initiative on Biodiversity, the European Green Deal, and emphasised by the Intergovernmental Science-Policy Platform on Biodiversity and Ecosystem Services (IPBES, https://ipbes.net/), the Convention on Biological Diversity (CBD, https://www.cbd.int/), and COP15. This was recently echoed also by the PNRR, which aims at realizing an ecological transition in Italy.

Following Minelli’s (2013) vision, such a repository should primarily focus on the preservation and valorisation of natural history collections, assuring at the same time a role of coordination and service. On the other hand, the activity of science dissemination through exhibition activities could be left to the existing local museums which are closer to citizens. This would help realising an efficient museum network, going from a local to a national level. This would also enable the development of a national strategy in biodiversity research, taking advantage of existing collections, with a particular focus on taxonomic studies, the baseline for any future conservation effort. Here we quote some of the core activities that could be carried out by the repository:

- ***Collection preservation.*** A repository assures that the preserved material obtained through field surveys, purchases, and salvages of dead animals is not lost. In fact, local museums often privilege the reception of collections with relevant ostensive and historical value and may discard collections with a mere scientific value. Moreover, private collections (particularly of entomology, ornithology, malacology, and herbaria) are often destroyed after the death of the owner: potentially, these collections are a huge treasure of biodiversity that may be acquired by museums free of charge, but they are often refused due to lack of space. Part of the types of newly described taxa (mainly from Italy) could also find space in such a repository, as well as samples collected during ecological studies (Pyke and Ehrlich 2010).
- ***Museums coordination.***Minelli (2013, 2015) and Andreone et al. (2014) already highlighted the need of a “metamuseum” or “diffused museum” connecting the small and medium-sized Italian museums. The biorepository hub could also be done under national coordination to assure the management of the existing natural history collections.
- ***Collection digitalization.*** Most of the Italian collections still need be digitalized. This would contribute to the creation of a “meta-catalogue” to allow rapid check of vouchers. This task would benefit from an accompanying photographic collection, especially of all of type specimens, including CT scans of remarkable specimens (types and rare or extinct species) and other kinds of data, i.e., bio-acoustics.
- ***Shared services for taxonomy.*** The biorepository could also support specific taxonomic, phylogenetic, and ecological studies, providing services and expertise. It could act as a central storage hub for genetic samples responding to the highest standards of research (e.g., Phillips et al. 2019), by assuring the formation of a scientific staff for a wide range of disciplines and following field-collecting policies based on established priorities. Hired taxonomists could also provide services to local museums, institutions, and other organizations that need biodiversity information. Collections and expertise could be utilised in the monitoring of alien species at the national level and in the identification of traded species (Palandačić et al. 2020).
- ***Taxonomic training.*** The expertise on taxonomy is fading away (Boero 2010), and the training of new taxonomists mastering both morphological and molecular approaches is crucial to appreciate the state of the natural capital. The organization of courses on biodiversity given by the few remaining specialists might be a crucial enterprise of the NBFC, to revive the Italian expertise in taxonomy, as done by the National Science Foundation of the US with the Partnership to Enhance Expertise in Taxonomy (Boero 2001).
- ***Sanitary observatory.*** In collaboration with sanitary authorities, the centre would be the ideal place to deal with biosurveillance activities through the storage of critical specimens that, through an efficient tracking system, are associated with identified pathogens of potential health significance (Thompson et al. 2021). The repository would also serve as location not only for primary vouchers but also for secondary and complementary vouchers of, for example, parasites and pathogens (Colella et al. 2021).

## ﻿A plea for a new model of research centre

Summing up, the historical fragmentation of Italian museums, and the manifest difficulties in managing and promoting the housed scientific collections, call for a novel concept of a centralised research centre. The global ecological crisis, the availability of PNRR funds, and the urgency to realise an ecological transition focusing on the integrity of the natural capital in terms of biodiversity and ecosystems can contribute to the birth of a new model of coordination and a research centre that could make up for the lack of a national museum of natural history. A centralised repository and a new collaboration of the existing museological institutions is necessary and urgent: the NBFC is a unique and unrepeated occasion for such a development.

The collection of biological vouchers and the easily accessible availability of natural history collections connected in an operative network within Italy, as well as to researchers from all over the world, is also a priority, particularly for future generations (Rohwer et al. 2022). The existing system of museums should work in a coordinated way to develop a web of institutions, with an interaction from the largest museums to the smallest ones, providing shared resources and facilities. In particular, a centralised research centre could coordinate the research activities led by Italian museums, i.e., providing instruments and tools that are currently missing. This technological supply could allow the realisation of a real hub with efficient interconnections with the existing museums managed by disparate institutions and administrations.

The funds from PNRR are vital to recruit curatorial and scientific personnel that makes the hub the national taxonomy focal point, supporting an organic nationwide action of voucher digitalisation, a high-quality photographic catalogue of most of the specimens (with special emphasis to types), and collaborating with all national biodiversity-related initiatives and bodies (the national protected area system, Italian fauna checklist project, invasive species monitoring, etc). The physical location of the repository, if it will be newly realised or obtained by empowering an already existing institution, obviously depends on a series of choices, including economic and political considerations, which are outside the primary scope of the present paper.

The definition and identification of a biorepository and biodiversity hub will also give strength to the international commitments agreed upon by the Italian government under the Convention on Biological Diversity and the upcoming targets of the Post-2020 Global Biodiversity Framework, where knowledge and conservation of each nation’s biodiversity will be at the core of the goals. When this happens, Italy will be endowed with a large and efficient institution for the knowledge and conservation of biodiversity, and a crucial structure to assure a true ecological transition.
